# Supplementary figures and images for: To integrate or not to integrate: Temporal dynamics of hierarchical Bayesian causal inference
Source: PLoS Biol. 2019 Apr 2;17(4):e3000210. doi: 10.1371/journal.pbio.3000210 (PMC6461295; doi:10.1371/journal.pbio.3000210)

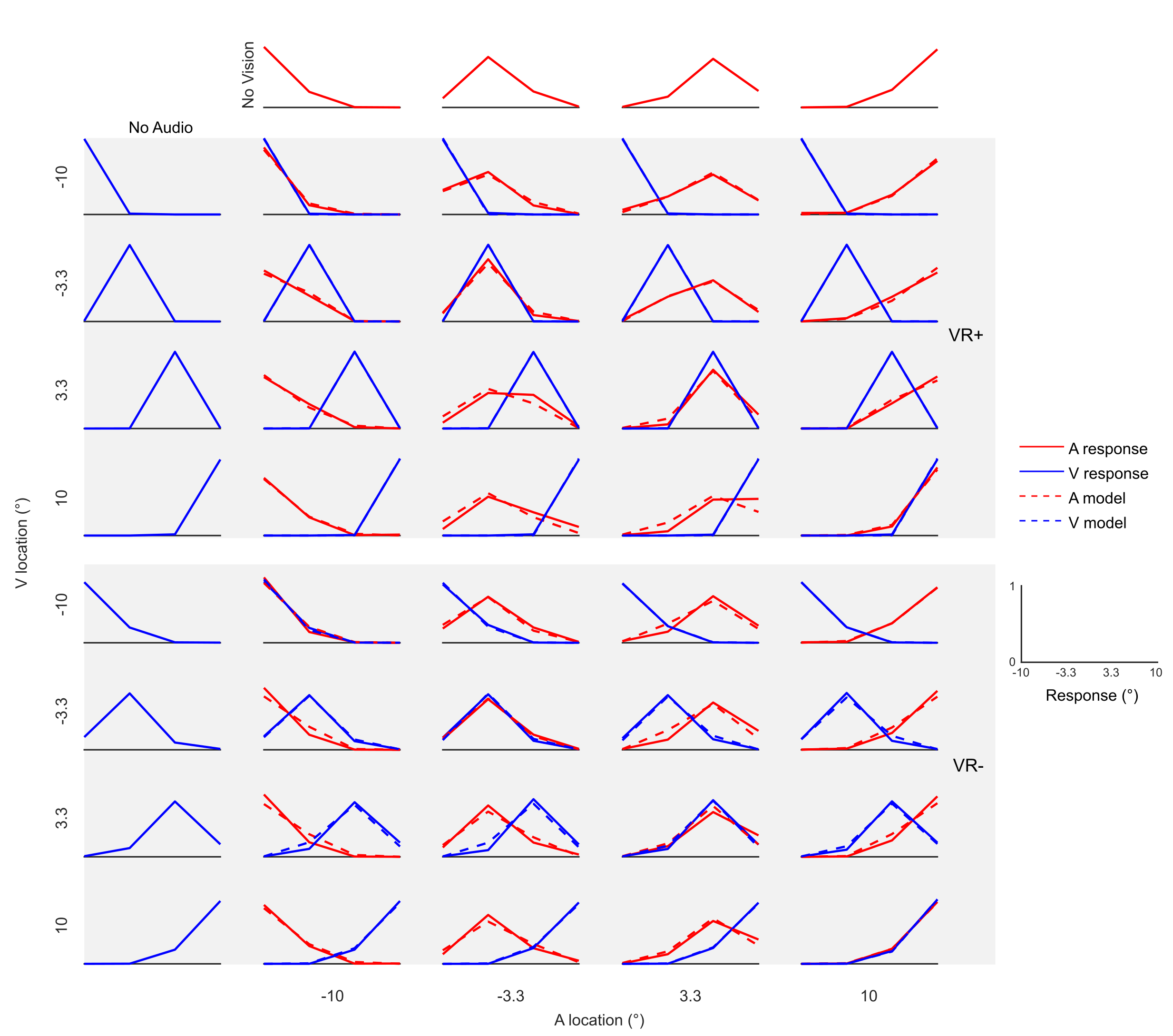

Supplement: S1 Fig — The distribution (across participants’ mean) of spatial estimates given by observers’ behavioural localisation responses (solid lines) or predicted by the Bayesian causal inference model fitted to observers’ behavioural responses (dashed lines, for model averaging) are shown across all conditions in our 2 task relevance (auditory: red versus visual: blue) × visual reliability (high: row 1–4 versus low: row 5–8) × 4 auditory location (columns as indicated) × 4 visual location (rows as indicated) design. (TIF) [file pbio.3000210.s003.tif]

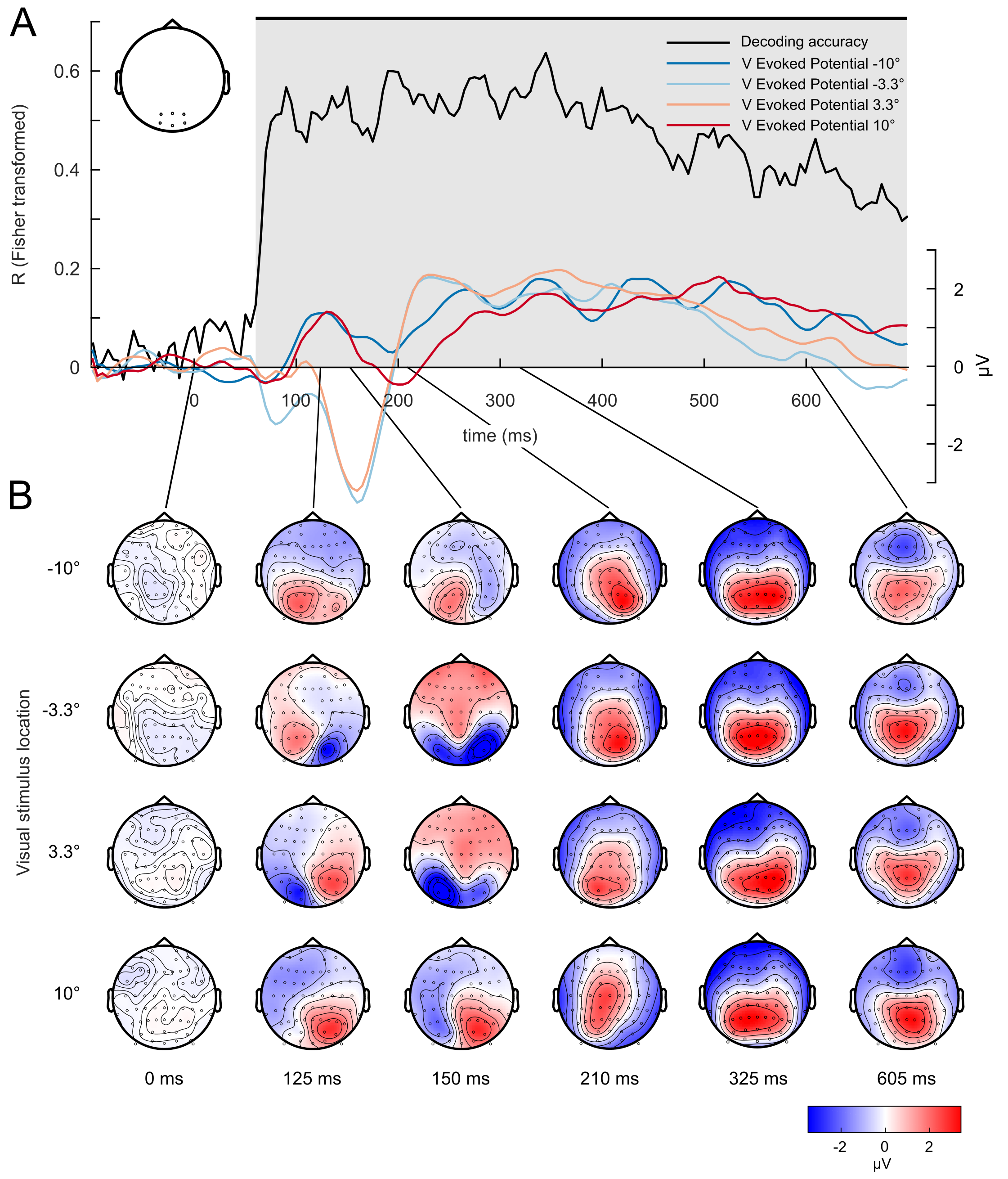

Supplement: S2 Fig — (A) Time course of decoding accuracy (i.e., Pearson correlation between true and predicted visual stimulus locations pooled over both visual reliabilities, black line) and the EEG evoked potentials (across participants’ mean) for the unisensory visual (high reliability only) signals at −10°, −3.3°, 3.3°, and 10°, averaged over occipital channels. Shaded grey area indicates the time window at which the decoding accuracy is significantly better than chance. EEG signals were averaged across the electrodes shown in the inset. (B) EEG topographies (across participants’ mean) for the unisensory visual signals (high reliability only) at −10°, −3.3°, 3.3°, and 10° shown at the given time points. (TIF) [file pbio.3000210.s004.tif]

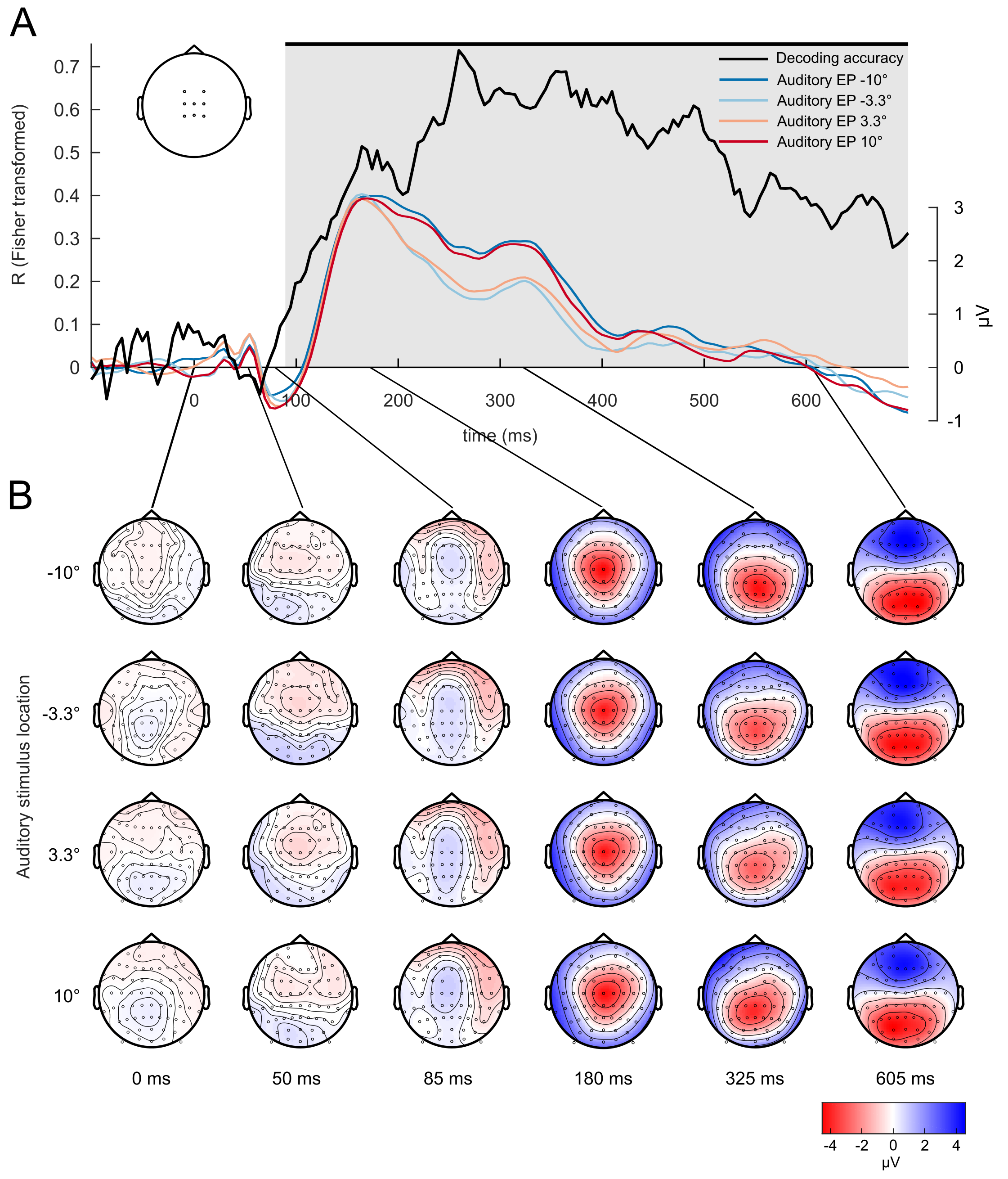

Supplement: S3 Fig — (A) Time course of decoding accuracy (i.e., Pearson correlation between true and predicted stimulus locations, black line) and the EEG evoked potentials (across participants’ mean) for the unisensory auditory signals at −10°, −3.3°, 3.3°, and 10°, averaged over central channels. Shaded grey area indicates decoding accuracy significantly better than chance. EEG signals were averaged across the electrodes shown in the inset. (B) EEG topographies (across participants’ mean) for the unisensory auditory stimuli at −10°, −3.3°, 3.3°, and 10° shown at the given time points. (TIF) [file pbio.3000210.s005.tif]

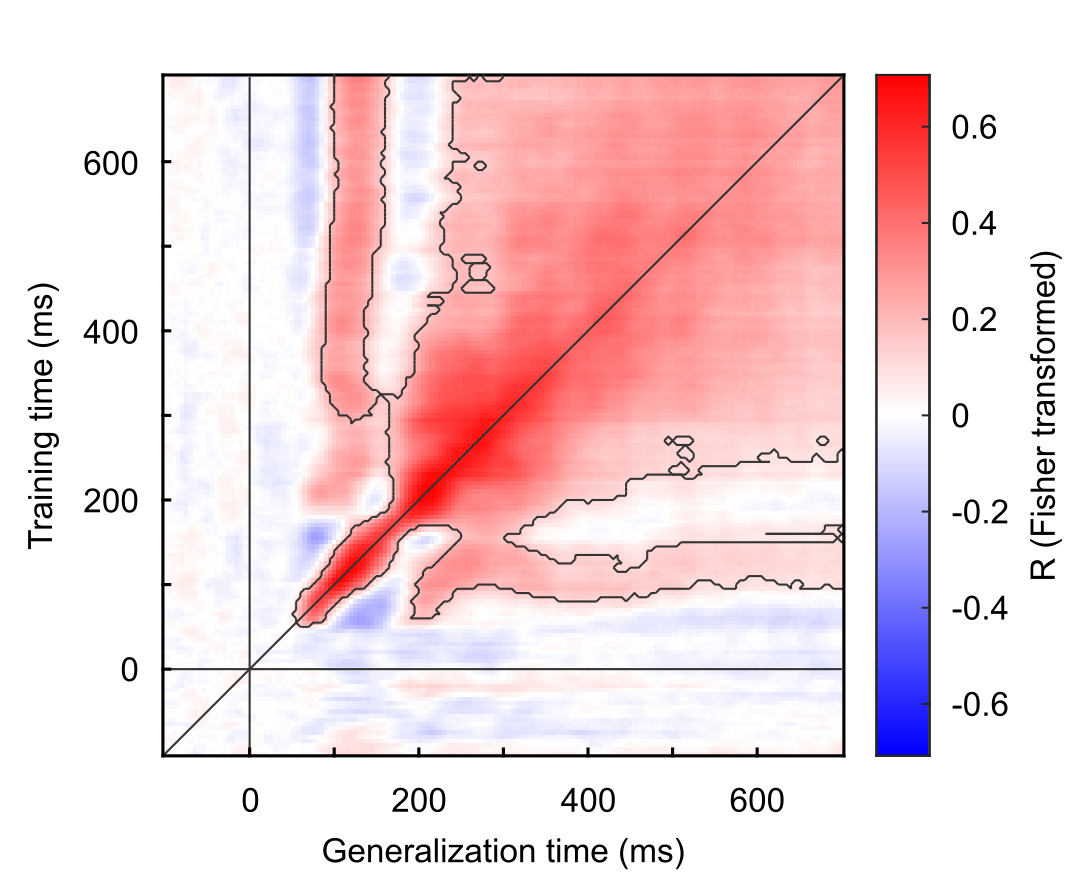

Supplement: S4 Fig — The temporal generalisation matrix shows the decoding accuracy for audiovisual congruent trials across each combination of training (y-axis) and testing (x-axis) time point. The grey line along the diagonal indicates where the training time is equal to the testing time. Horizontal and vertical grey lines indicate the stimulus onset. The thin black lines encircle the cluster with decoding accuracies that were significantly better than chance at p < 0.05 corrected for multiple comparisons. (TIF) [file pbio.3000210.s006.tif]

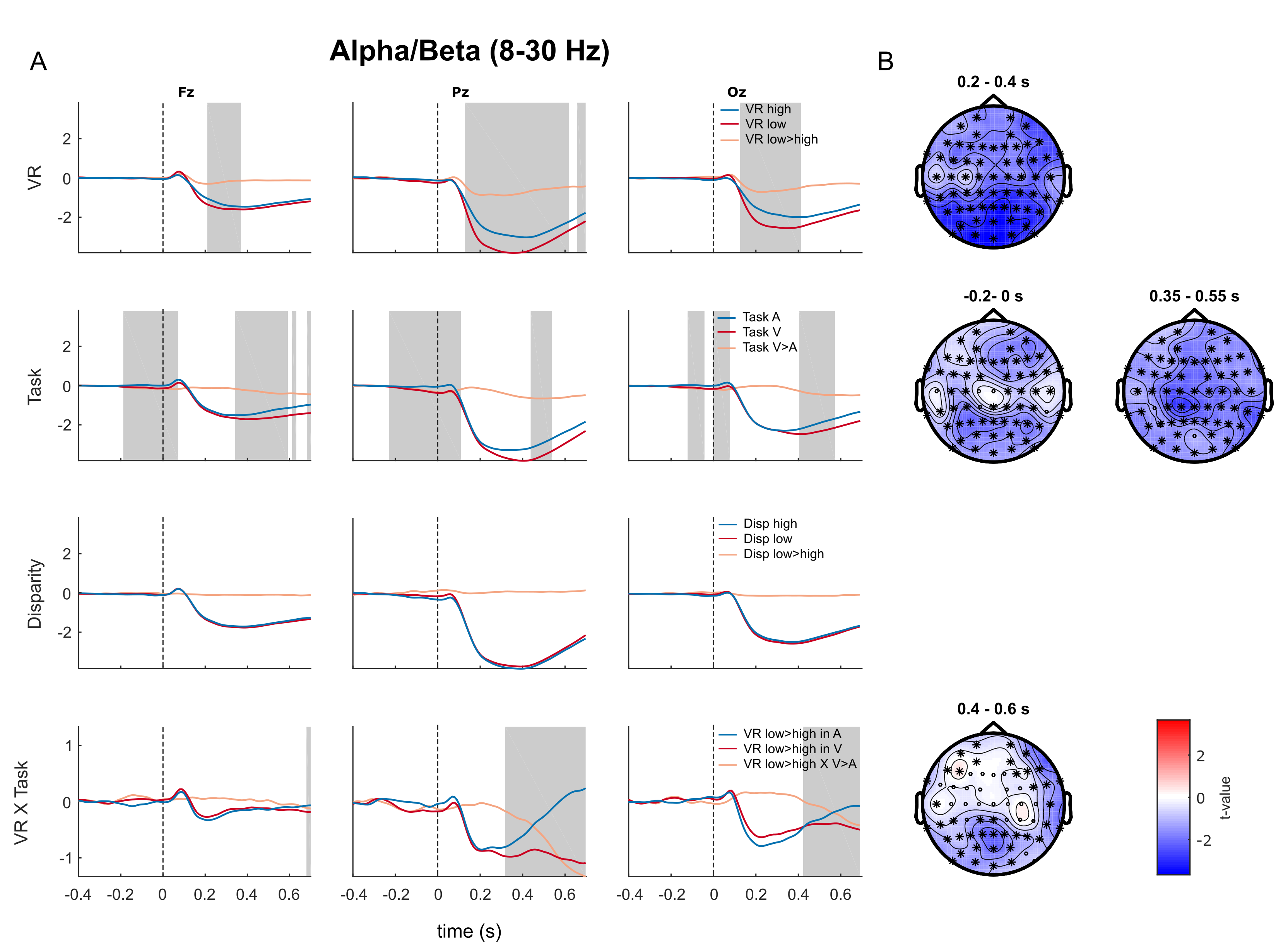

Supplement: S5 Fig — (A) Time courses of total power averaged over the alpha/beta (8–30 Hz) frequency bands (baseline corrected using prestimulus window [−400 ms to −200 ms]) are shown for the main effects of visual reliability (row 1), task relevance (row 2), spatial disparity (row 3), and the visual reliability × task relevance interaction (row 4) at three selected electrodes (i.e., Fz = left; Pz = middle; Oz = right columns). For each effect, we show the power for the difference (or interaction) and the individual conditions coded in different colours as indicated for each row. Grey shaded areas indicate the time windows where at least one electrode was part of the significant cluster after correcting for multiple comparisons across time (i.e., −200 ms to 700 ms), frequency (i.e., 4–30 Hz), and topography. (B) Topographies of the t values averaged across the significant time windows of the corresponding effects. Electrodes marked with black stars were part of the significant cluster (corrected across topography × time × frequency). (TIF) [file pbio.3000210.s007.tif]
