# Supplementary material for: To integrate or not to integrate: Temporal dynamics of hierarchical Bayesian causal inference
Source: PLoS Biol. 2019 Apr 2;17(4):e3000210. doi: 10.1371/journal.pbio.3000210 (PMC6461295; doi:10.1371/journal.pbio.3000210)
Supplement: S1 Table — Model averaging (BCIavg), model selection (BCIsel), and probability matching (BCImatch). BCI, Bayesian causal inference; PEP, protected exceedance probability; R2, coefficient of determination; relBICgroup, group-level relative Bayesian information criterion [25]. (DOCX) [file pbio.3000210.s008.docx]

**S1 Table**

|  | p_c_ | σ_p_ | σ_A_ | σ_V1_ | σ_V2_ | R^2^ | relBIC_group_ | PEP |
| --- | --- | --- | --- | --- | --- | --- | --- | --- |
| BCI_avg_ | 0.15 ± 0.04 | 36.4 ± 11.0 | 4.4 ± 0.2 | 0.3 ± 0.15 | 3.5 ± 0.24 | 0.857 ± 0.003 | 0 | 0.784 |
| BCI_sel_ | 0.28 ± 0.04 | 25.8 ± 5.3 | 4.4 ± 0.2 | 0.3 ± 0.16 | 3.5 ± 0.23 | 0.856 ± 0.003 | -60.5 | 0.113 |
| BCI_match_ | 0.15 ± 0.03 | 33.0 ± 9.4 | 4.1 ± 0.2 | 0.3 ± 0.16 | 3.4 ± 0.23 | 0.856 ± 0.003 | -54.8 | 0.103 |
