# Supplementary material for: To integrate or not to integrate: Temporal dynamics of hierarchical Bayesian causal inference
Source: PLoS Biol. 2019 Apr 2;17(4):e3000210. doi: 10.1371/journal.pbio.3000210 (PMC6461295; doi:10.1371/journal.pbio.3000210)
Supplement: S2 Table — Significant effects are shown for overall relative to baseline, main effect of VR, and main effect of task relevance (‘Task’), and the interaction between VR and task relevance is shown across rows. Columns of the table indicate the approximate time windows that the significant cluster spanned. All p-values are reported at the cluster level, corrected for multiple comparisons over time × topography × frequency. VR, visual reliability. (DOCX) [file pbio.3000210.s009.docx]

**S2 Table**

|  | Neural latency | | | | |
| --- | --- | --- | --- | --- | --- |
| Effect | ~ -200 ms | ~ 50 ms | ~ 100 ms | ~ 200 ms | ~ 400 ms |
| Overall |  | α/β 50 – 100 ms (p = 0.054) | α/β 100 - 700 ms (p = 0.0001) | | |
| VR |  |  |  | α/β 200 - 400 ms (p = 0.0001) | |
| Task | α/β -200 – 0 ms  (p = 0.013) |  |  | α/β 350 – 550 ms  (p = 0.013) | |
|  | γ -200 – 700 ms (p = 0.0002) | | | | |
| VR X Task |  |  |  |  | α/β 400 - 700 ms (p = 0.005) |
